# Supplementary figures and images for: Intracellular acidification is a hallmark of thymineless death in E. coli
Source: PLoS Genet. 2022 Oct 24;18(10):e1010456. doi: 10.1371/journal.pgen.1010456 (PMC9632930; doi:10.1371/journal.pgen.1010456)

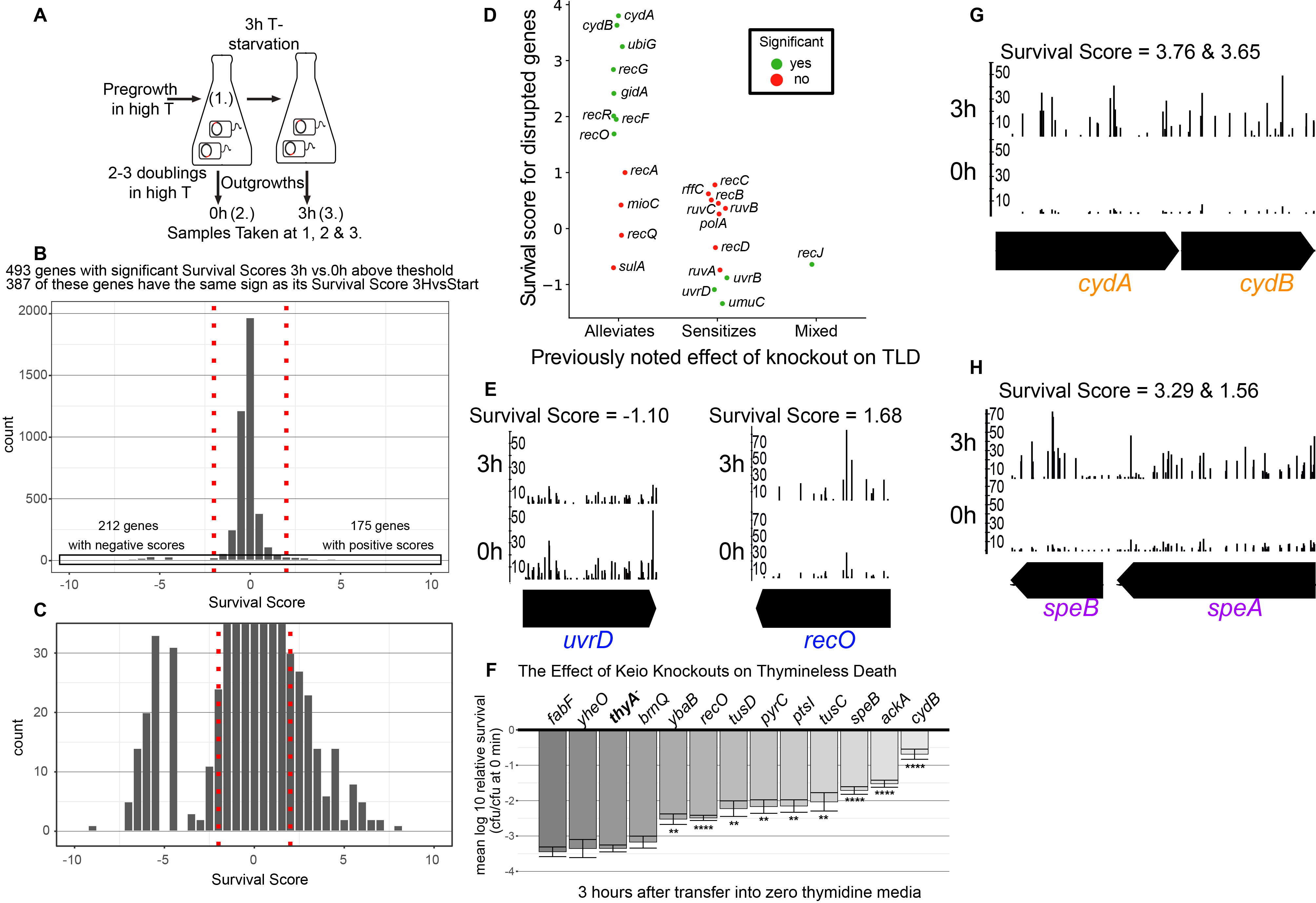

Supplement: S1 Fig — (A) Survival profiling time points, with and without outgrowths (see Methods). At 0h starvation and 3h starvation, an aliquot of the transposon library was placed in high thymidine for 2–3 doublings in order to amplify signal from living cells over residual signal from dead cells. An aliquot was also taken at the start of the selection, before the 0h outgrowth. (B) The distribution of survival scores and generation of candidate lists. 493 genes were above a threshold log2 fold change magnitude (shown by red dashed line) and had a q-value<0.05. Significance was calculated on the ratio of FPM at 3h vs. 0h using the rate ratio test and was corrected using the Benjamini & Yekutieli method. Any genes on this list that had an opposite sign from the survival score for the 3h vs. “Start” were discarded, yielding a total of 387 candidate genes. (C) is an inset for the area pictured in the rectangle in panel (B). (D) Survival scores (and significance) for genes previously known to have effects on TLD survival when knocked out. (E; G-H) Transposon insertions visualized using the Integrated Genome Browser. The frequency of each insertion site is shown in read counts per million. (E) Frequency of transposon insertions along the length of two previously known TLD contributors at 0h and after 3h thymidine starvation. A deletion in uvrD is known to sensitize cells to TLD and a deletion in recO is known to alleviate TLD. (F) Candidates were validated by transferring Keio collection knockout alleles into MG1655 thyA-, and assessing their survival at 3h of thymidine starvation. All death assays, unless otherwise stated, were performed at 37°C. Relative survival was measured for at least three independent experiments, with error bars representing standard error of the mean. p-values for all death assays were calculated using a Welch t-test. * P<0.05, ** P<0.01, *** P < 0.001, **** P<0.0001. (G) Enhancements of transposon insertions within the cydAB operon. (H) Enhancements of transposo [file pgen.1010456.s001.tiff]

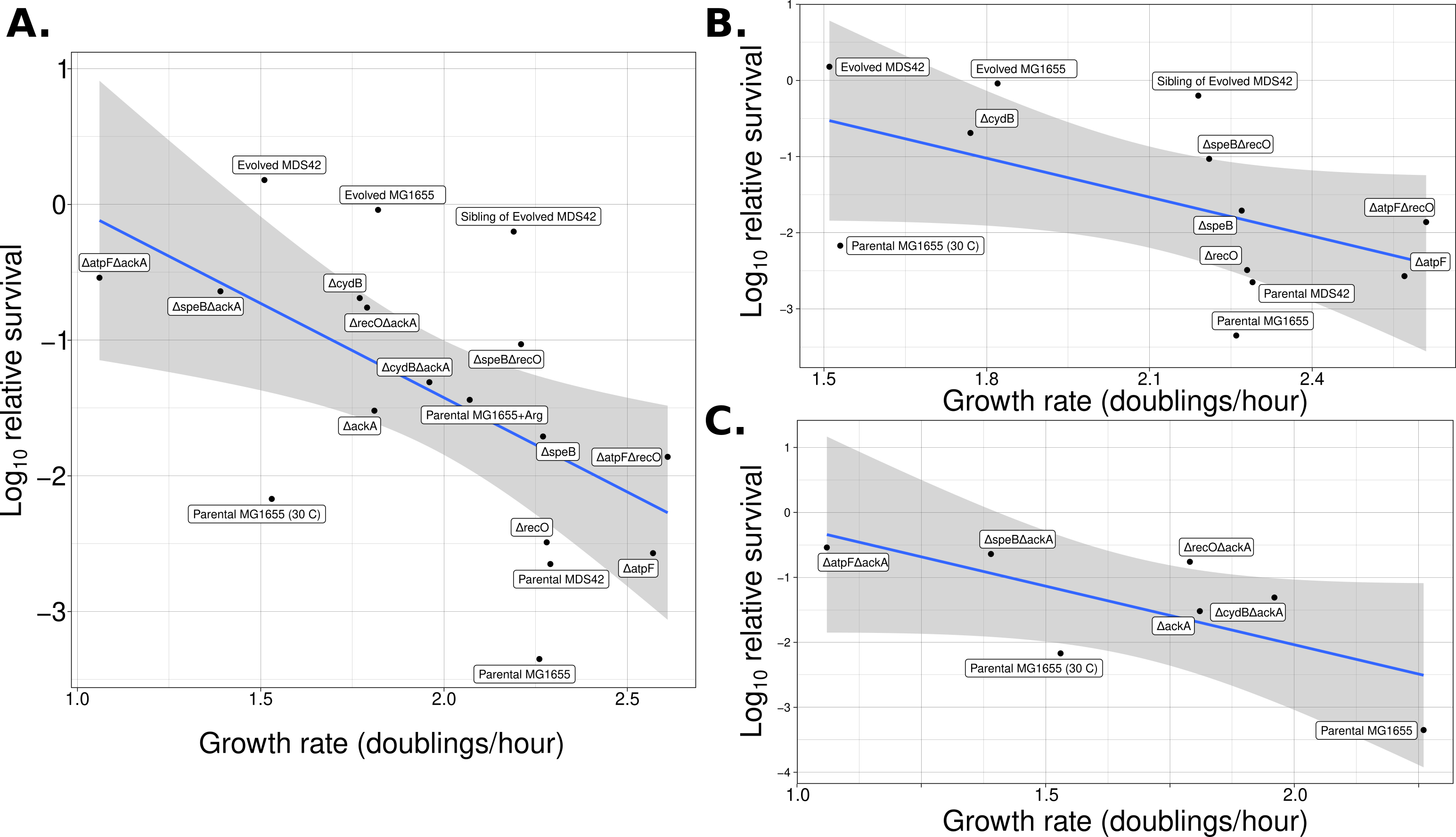

Supplement: S2 Fig — A. All strains. B. All strains minus the ackA strains. C. ackA strains only. See Methods under Growth Rate and Death Assays. (TIFF) [file pgen.1010456.s002.tiff]

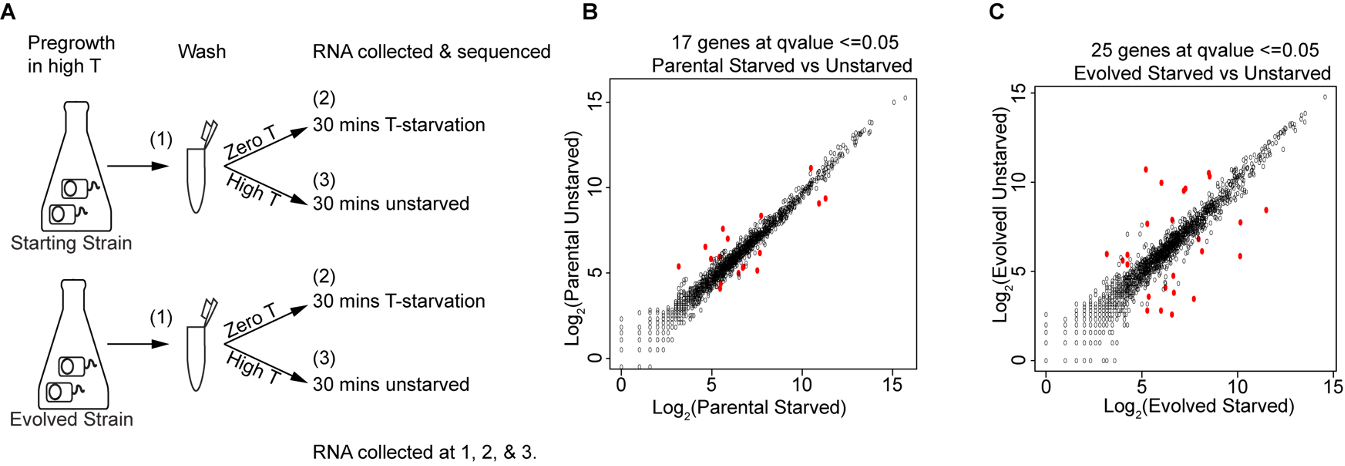

Supplement: S3 Fig — (A) Experimental setup of RNA sequencing of parental and evolved strains in the MDS42 background. All steps were performed at 30°C (the temperature at which laboratory evolution was conducted). Pregrowth was for 2h, and RNA was collected both before pelleting (1) and 30 minutes after pelleting and transfer into thymidine-free media (2). RNA was also collected 30 minutes after pelleting and placement back in high thymidine as a control (3). (B-C) Log2 expression of genes in two comparisons showing significant differentially expressed genes in red. The 17 and 25 significant differentially expressed genes can be found in S4D and S4C Fig, respectively. (B) Parental starved vs. unstarved. (C) Evolved starved vs. unstarved. (TIFF) [file pgen.1010456.s003.tiff]

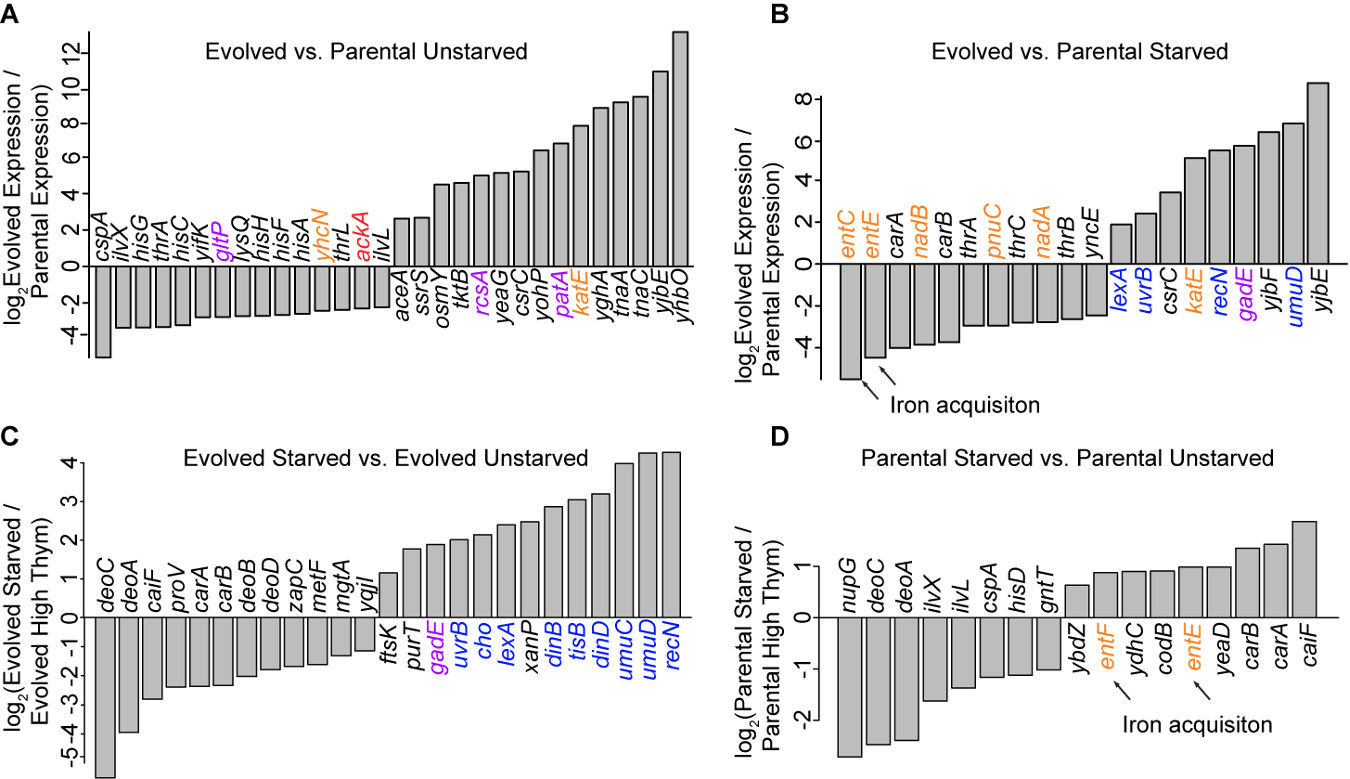

Supplement: S4 Fig — (A-D) Significant differentially expressed genes in various comparisons. See S8 Table for the q-values. The LFCs of RPKMs are visualized here. (A) Significant differentially expressed genes in the evolved vs. the parental in the unstarved condition. (B) Significant differentially expressed genes in the evolved vs. parental 30 minutes into thymidine starvation. (C) Significant differentially expressed genes in the evolved starved vs. evolved unstarved. (D) Significant differentially expressed genes in the parental starved vs. parental unstarved. As in the main text, purple represents genes involved in putrescine / glutamate / arginine metabolism or their associated amino acid decarboxylation acid resistance systems; orange represents genes involved in ROS; red represents genes involved in acetate dissimilation; and blue represents genes involved in DNA replication/repair. (TIFF) [file pgen.1010456.s004.tiff]

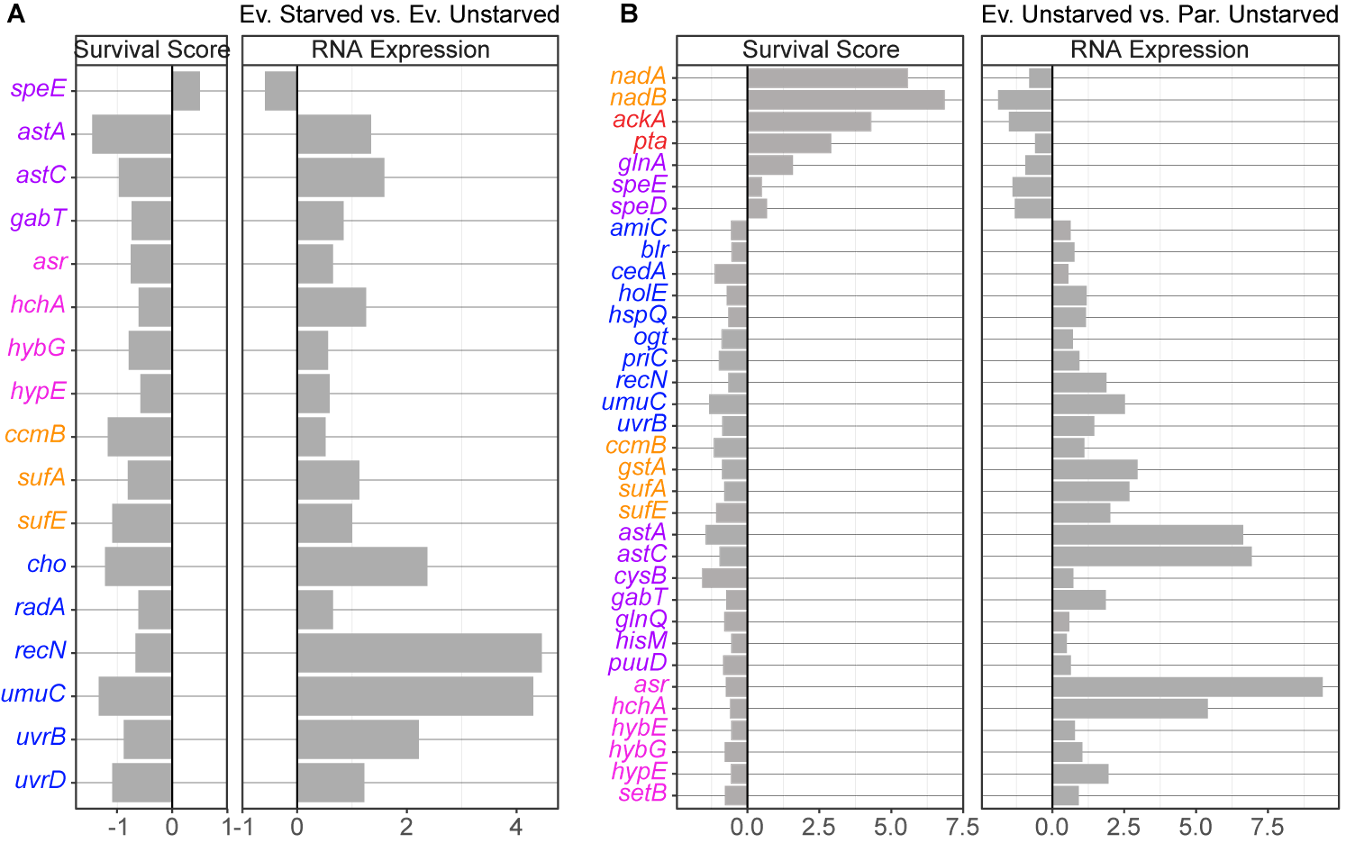

Supplement: S6 Fig — (A-B) The left-hand column shows survival scores from the survival profiling experiment and the right-hand column shows LFC of mRNA expression (TPM) from the transcriptome profiling experiment. (A) Survival scores with the LFC of RNA expression in evolved starved vs. evolved unstarved. The remaining genes showing concordant effects can be found in S10 Table. (B) Survival scores with the LFC of RNA expression in evolved unstarved vs. parental unstarved. The remaining genes showing concordant effects can be found in S11 Table. Colors are used to represent recurring pathways as described in S6 Fig. Pink represents genes involved in proton translocation (or sequestration) systems or in the acid stress response. (TIFF) [file pgen.1010456.s006.tiff]

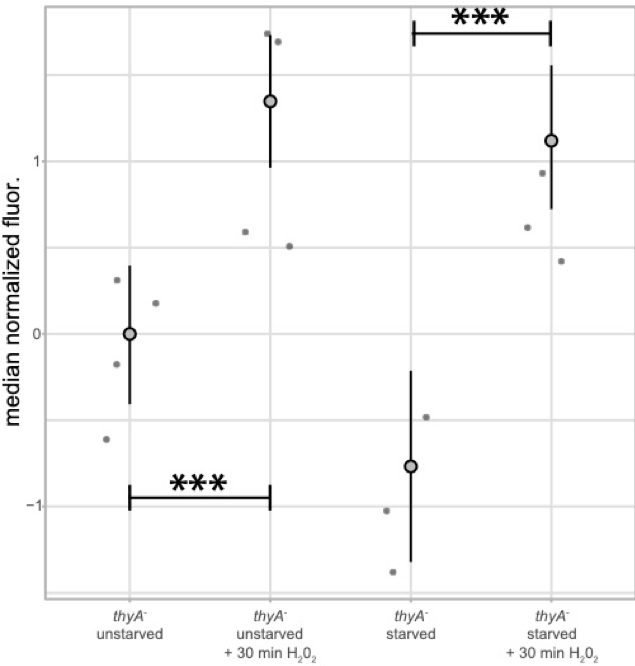

Supplement: S9 Fig — Adjusted fluorescence measured using flow cytometry of thyA- cells stained with Peroxy Orange with and without a 30-minute incubation with 1mM H202. The two plots on the left show adjusted fluorescence at 1.5h in high thymidine media and the two plots on the right show adjusted fluorescence at 1.5h thymidine starvation. See Fig 3 caption for definitions of plotted intervals and significance tests. (TIFF) [file pgen.1010456.s009.tiff]

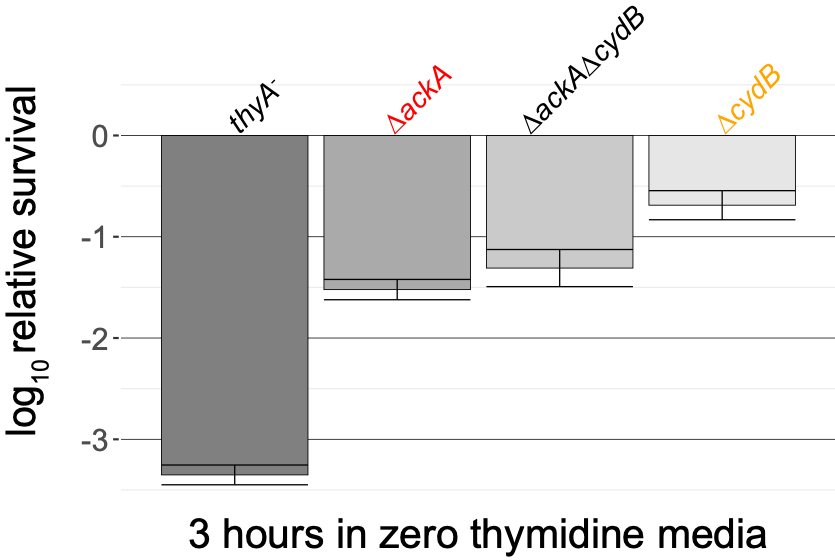

Supplement: S11 Fig — Survival of the parental strain and knockouts in the MG1655 background at 3h thymidine starvation. Relative survival was measured for at least three independent experiments, with error bars representing standard error of the mean. (TIFF) [file pgen.1010456.s011.tiff]
