# Supplementary material for: Intracellular acidification is a hallmark of thymineless death in E. coli
Source: PLoS Genet. 2022 Oct 24;18(10):e1010456. doi: 10.1371/journal.pgen.1010456 (PMC9632930; doi:10.1371/journal.pgen.1010456)
Supplement: S4 Table — (PDF) [file pgen.1010456.s018.pdf]

locus	TLD phen relative to wt	reference	Survival Score
ruvABC	sensitizes	(1), (2)	-0.74; 0.36; 0.45
recBCD	sensitizes	(3), (2), (4)	0.51; 0.78; -0.34
uvrD	sensitizes	(3), (2), (5)	-1.09
polA1	sensitizes	(6)	0.26 *(polA)*
umuC	sensitizes	(7)	-1.34
uvrB	sensitizes	(8)	-0.88
rffC	sensitizes	(9)	0.62
recJ	mixed results	(1, 2, 10)	-0.64
sulA	alleviates	(1), (11)	-0.70
recA	alleviates	(1), (2)	1.0
recG	alleviates	(2)	2.84
recF	alleviates	(1), (2), (12), (13)	1.95
recQ	alleviates	(3), (14), (13)	-0.12
recO	alleviates	(2), (10), (13)	1.69
recR	alleviates	(1, 2, 12)	2.01
mioCp9	alleviates	(15)	0.42 (*mioC)*
PmioCPgid	alleviates	(16)	2.38 (*gidA*)
cydA	alleviates	(17)	3.80
ubiG	alleviates	(18)	3.25
cydB	alleviates	(18)	3.63

Genes in blue show significant effects in our Survival Profiling screen.

1. Fonville NC, Bates D, Hastings PJ, Hanawalt PC, Rosenberg SM. Role of RecA and the SOS response in thymineless death in Escherichia coli. PLoS Genet. 2010;6(3):e1000865.
2. Kuong KJ, Kuzminov A. Stalled replication fork repair and misrepair during thymineless death in Escherichia coli. Genes Cells. 2010;15(6):619-34.
3. Fonville NC, Vaksman Z, DeNapoli J, Hastings PJ, Rosenberg SM. Pathways of resistance to thymineless death in Escherichia coli and the function of UvrD. Genetics. 2011;189(1):23-36.

4. Martin CM, Guzman EC. DNA replication initiation as a key element in thymineless death. DNA Repair (Amst). 2011;10(1):94-101.
5. Siegel EC. Ultraviolet-sensitive mutator strain of Escherichia coli K-12. J Bacteriol. 1973;113(1):145-60.
6. Berg CM, O'Neill JM. Thymineless death in polA+ and polA- strains of Escherichia coli. J Bacteriol. 1973;115(2):707-8.
7. Rao TVP, Kuzminov A. Sources of thymidine and analogs fueling futile damage-repair cycles and ss-gap accumulation during thymine starvation in Escherichia coli. DNA Repair (Amst). 2019;75:1-17.
8. Slezarikova V, Sedliakova M. uvrB-dependent, recF-independent post-replication (or replication) repair in Escherichia coli. J Photochem Photobiol B. 1991;10(4):329-37.
9. Rao TVP, Kuzminov A. Oxidative Damage Blocks Thymineless Death and Trimethoprim Poisoning in Escherichia coli. J Bacteriol. 2022;204(1):e0037021.
10. Nakayama K, Shiota S, Nakayama H. Thymineless death in Escherichia coli mutants deficient in the RecF recombination pathway. Can J Microbiol. 1988;34(7):905-7.
11. Hamilton HM, Wilson R, Blythe M, Nehring RB, Fonville NC, Louis EJ, et al. Thymineless death is inhibited by CsrA in Escherichia coli lacking the SOS response. DNA Repair (Amst). 2013;12(11):993-9.
12. Nakayama H, Nakayama K, Nakayama R, Nakayama Y. Recombination-deficient mutations and thymineless death in Escherichia coli K12: reciprocal effects of recBC and recF and indifference of recA mutations. Can J Microbiol. 1982;28(4):425-30.
13. Sangurdekar DP, Hamann BL, Smirnov D, Srienc F, Hanawalt PC, Khodursky AB. Thymineless death is associated with loss of essential genetic information from the replication origin. Mol Microbiol. 2010;75(6):1455-67.
14. Nakayama H, Nakayama K, Nakayama R, Irino N, Nakayama Y, Hanawalt PC. Isolation and genetic characterization of a thymineless death-resistant mutant of Escherichia coli K12: identification of a new mutation (recQ1) that blocks the RecF recombination pathway. Mol Gen Genet. 1984;195(3):474-80.
15. Bouvier F, Sicard N. Interference of dna ts mutations of Escherichia coli with thymineless death. J Bacteriol. 1975;124(3):1198-204.
16. Martin CM, Viguera E, Guzman EC. Rifampicin suppresses thymineless death by blocking the transcription-dependent step of chromosome initiation. DNA Repair (Amst). 2014;18:10-7.
17. Strauss B, Kelly K, Ekiert D. Cytochrome oxidase deficiency protects Escherichia coli from cell death but not from filamentation due to thymine deficiency or DNA polymerase inactivation. J Bacteriol. 2005;187(8):2827-35.
18. Hong Y, Li L, Luan G, Drlica K, Zhao X. Contribution of reactive oxygen species to thymineless death in Escherichia coli. Nat Microbiol. 2017;2(12):1667-75.
