# Supplementary material for: Intracellular acidification is a hallmark of thymineless death in E. coli
Source: PLoS Genet. 2022 Oct 24;18(10):e1010456. doi: 10.1371/journal.pgen.1010456 (PMC9632930; doi:10.1371/journal.pgen.1010456)
Supplement: Attachment — Submitted filename: Response to the Reviewers Sep2_2022-final.pdf [file pgen.1010456.s026.pdf]

We thank the reviewers for their insightful comments and feedback. We have substantially revised the manuscript in careful consideration of all reviewer assessments. In addition, we have performed/ included critical additional experiments and analyses that now substantially improve the exposition and the support for our conclusions, and solidify our findings. These additional experiments and analyses include:

- New column to S1 Table adding an additional test for high-confidence hits from our Survival Profiling experiment in order to provide an additional layer of statistical confidence
- New panel D in S1 Fig which shows that all of the genes for which prior information in the literature exists regarding TLD survival and for which we observed significant fitness scores, show fitness scores consistent with the previously demonstrated survival effects and a new S4 Table to accompany this figure
- New panel F in S1 Fig (with accompanying new S4 Table) to show that our false positivity rate for our survival profiling (Tn-Seq) is low
- New figure S5 to show the enrichment in genes with consistent responses vs. non-consistent responses between our survival profiling and transcriptome profiling experiments
- New figure S7 to show kill curves for the most sensitive strains before, at, and after the chosen flow cytometry time points to establish why these specific time points are appropriate and the data collected at them instructive;
- New panel S10A for a new flow experiment that sorted an isogenic population of cells into the top and bottom tails of the pH-sensitive fluorescence distribution to show that cells with higher acidification have lower survival; new panel S10B is the accompanying control that establishes that the dyes do not affect survival.
- New figure S13 to establish that at the time point at which we are seeing significant cytoplasmic acidification changes for the gentamycin treated cells (and no ROS accumulation) we are seeing a low amount of death.

Below we address all the concerns of the three reviewers, point-by-point.

Reviewer #1: The study uses three complementary approaches to examine the TLD phenomenon in E. coli:
1. Tn-Seq profiling to find genes that enhance survival during thymidine starvation
2. Laboratory evolution to find mutants with TLD resistance
3. Transcriptomics from thymidine-starved and non-starved ancestral and evolved strains to identify genes that are differently affected by thymidine starvation in the evolved vs. the ancestral strains

Aside from identifying genes known from previous studies to be involved in TLD, the study identifies genes directly or indirectly involved in pH homeostasis as part of TLD. The data from the Tn-Seq, evolution experiments, and transcriptomics are solid and complements each other very nicely.

I have no major concerns, and only two comments that I think could improve the manuscript:

Comment #1: The use of flow cytometry to measure fluorescence of bacteria has been shown to be prone to artefacts due to changes in cells size caused by e.g. treatment with various antibiotics. This has been addressed and is described in the Methods section, but I think it could also be mentioned in appropriate places in the results section (and perhaps discussed as part of addressing Comment #2).

We thank this reviewer for their insightful comments. Given that much of the previous flow cytometry work in this field has not done these important normalizations, we regret that we did not previously emphasize this in the main text. In the revised manuscript, we now emphasize in the results section and the discussion that all the flow cytometry data has been normalized to account for any changes in cell size and other potential confounding artifacts.

Comment #2: The involvement of ROS in cell death during TLD is controversial (and connected to the even more controversial hypothesis that ROS is involved in a unifying killing mechanism shared by several different classes of bacteriocidal antibiotics). The ROS damage hypothesis in TLD was originally supported by experiments where the cells were treated with the Fe(II) chelator bipyridyl to block oxidative damage caused by Fenton's reaction. Bipiridyl prevented TLD and prevented the detection of ROS (as detected by flow cytometry in dye-labeled cells without correction for changes in cell size). However, more careful experiments indicated that the concentration of bipyridyl that was used actually inhibited growth, protecting against TLD by preventing DNA replication, and preventing the increase in fluorescence intensity of the dye-labeled cells by preventing cell growth. Use of another Fe(II) chelator that did not inhibit growth did not prevent TLD or increase ROS detection. An even more compelling blow against the ROS hypothesis in TLD was that adding exogeneous peroxide instead of aggravating TLD actually protected cells from TLD (for reference: https://journals.asm.org/doi/10.1128/JB.00370-21). I suggest the authors add a paragraph about this controversy to the discussion, with their arguments for why the ROS detection and pHi measurements reflect real changes in cell physiology that are part of the TLD phenomenon (perhaps including something about the risk of artefacts due to changes in cell size and shape and how to avoid it).

This is an excellent point. We now have three added paragraphs in the discussion that addresses this controversy and how our work avoids such artifacts as those described above. We feel our MS discussion is vastly improved thanks to this suggestion.

Reviewer #2: Thymineless death is an important phenomenon, resulting in rapid cell death in E. coli and other organisms. It has a long literature but lacks a complete understanding of its mechanistic underpinnings. In their manuscript "Intracellular acidification is a hallmark of thymineless death in E. coli", Ketcham, Freddolino, and Tavazoie use three genome scale technologies to try to dissect key additional features of the response, suggesting that intracellular acidification is a causal factor in thymineless death. Unfortunately, there are issues with their experiments, and additional experimentation and analyses are required to solidify their claims, as detailed below

1. The authors perform a Tn-seq screen to determine the effect of various E. coli knockouts on survival of a delta-thyA strain during thymidine starvation. I have concerns with both how the experiment was performed and with the data analysis.

a. The authors use a non-standard protocol for their Tn-seq experiment. Rather than collecting a sample at the onset of thymidine starvation and after 3 hrs of starvation, the authors collect these samples only after an additional 3 hrs of growth in high thymidine. Therefore, "hits" are those cells that resist TLD, and those cells that recover more rapidly. These may be different gene classes, perhaps explaining the plethora of hits.

The authors justify this non-standard protocol by saying that this will "amplify signal from live cells relative to any residual DNA from dead cells". If necessary, the authors could take several timepoints after initiation of thymineless death to determine which mutants are resistant, and then take timepoints

after re-addition of thymidine to make a further determination of which mutants show more rapid recovery.

We thank the reviewer for their thoughtful insights and suggestions. This reviewer makes a valid point that in an outgrowth setup like the one we deployed there is a danger in selecting for survivors that recover more rapidly. Transposon footprinting after a selection, as was performed here, requires the outgrowths and we used controls to avoid confounding effects. We believe the problem in fact lies in our failure to explain in clear language exactly how the hits were identified, as reviewer 3 also pointed out. After 3h thymidine starvation, the insertion library contained a mixed population of dead cells and survivors. Without the outgrowth in high thymidine media, there would be no way to identify which cells were alive after the thymidine starvation (due to contribution of DNA from dead cells). In other words, the footprinting would pick up the signal from both live and dead cells and both would be sequenced and mapped. Therefore, the outgrowths are critical to pick up the survivors. Dead cells that did not survive the thymidine starvation cannot undergo 2-3 doublings after high-T addition, as TLD is defined as the inability of T-starved cells to grow even after T-starvation has ended and thymidine is resupplied. The outgrowths are thus necessary to pick up the survivors. In order to control for any rapid growers during the high thymidine outgrowth, our survival scores are always calculated by normalizing the outgrowth after 3h thymidine starvation by an identical outgrowth performed at the 0h starvation time point (but, we stress, the durations of the outgrowths are the same in both cases). We have taken greater care to be explicit about this in the main text.

b. Additionally, taking multiple timepoints of the Tn-seq library under thymidine starvation without outgrowth (e.g. 1hr, 2hr, etc) would help the authors make many of their claims much more cleanly. For example, they could look at the length of time before the onset of TLD in addition to the rate of TLD in different classes of genes.

We agree that the selection of the Tn-seq library could be used for many different experiments and is a valuable resource that would allow us to obtain answers to different questions such as the length of time before TLD onset or the rate of death for different gene classes. Our goal here was to gauge how disruptions of all non-essential genes in the genome help or hurt survival during T-starvation, and then to cross reference these hits to other parallel approaches that gauge TLD survival. Also, it is important to stress that in order to perform these other experiments we would still need some way to separate the DNA of the survivors from the dead cells at 1hr, 2hr etc. At this point, however, taking additional timepoints would require a complete repeat of all of our experiments, and we believe that in light of the extensive follow-up experiments that we performed, additional repetition is at this point unnecessary.

Performing the Tn-seq screen in multiple ways (i.e. marginal thymidine concentration, drugs, etc.) would also provide additional insight and decrease the number of false positives they find.

We agree that many additional experiments could be performed on this Tn-seq library and that we could have extended our screen in many different and interesting ways, but because of the multi modal nature of our study, we were somewhat constrained in terms of the scope of this work. Our goal was to utilize various modalities to reinforce the pathways considered, and then to follow-up on candidates identified more thoroughly. In future work, we can clearly go further to include these more detailed

explorations of the selection conditions. It is important to stress, as we now take more pains to do, that our screen is a success. Many of the previously known players in TLD were identified, as were previously non-implicated genes in previously known pathways. Importantly, this screen was able to identify and validate genes and pathways not previously known to be involved. See more below for commentary on our (low) empirical false positive rate.

c. Was there a single replicate of the screen performed? The authors should at least do technical replicates of the screen to quantify the variability in their experimental protocol.

We agree that in most experiments, replicates are critically important, however in our extensive experience involving many different selection conditions using transposon profiling, we have seen excellent concordance between replicates. One reason is that since we have many transposon insertions in every gene (1 insertion every 27 base pairs), each transposon insertion is an independent measure of a perturbation and its fitness effect in an experiment. Since our analysis involves aggregating the effects of multiple transposons for each gene this gives us a highly robust measure. In addition, due to the multi modal nature of our design, we decided that this transposon profiling would serve as an initial screen from which we would then choose to validate a set of high-priority candidates.

We have now included an additional table and figure to show that the false positive rate for our survival profiling screen was quite low. Using the survival scores at 3h normalized for any growth effects and filtered for any inconsistencies in survival from a different time point (by choosing genes with LFC FPMs 3h/0h >1 and LFC FPMs 3h/Start >1), we generated a list of 52 candidates with positive survival scores (the new S4 Table). From this list, we generated 12 Keio knockouts in the MG1655 *thyA*$^-$ background, taking care to select genes across different pathways and GO Term categories. Nine of the 12 double mutants we generated and tested showed significantly increased survival from the *thyA*$^-$ at 3h T-starvation (the new S1F Fig), thus indicating an empirical false positive rate of 25% in our screen.

An additional robust approach used to filter out false positives from the candidates we focus on was to cross reference candidates from the survival profiling to those identified by other parallel approaches. Candidates from the survival profiling (Tn-seq) were cross-referenced to the candidates from transcriptome profiling (RNA seq) (Figure 2H&I and S5 Fig and Supplementary tables 7-9) – we focused on genes with concordant effects across experimental modalities (*i.e.*, genes with high fitness scores for transposon insertions that were also lower in expression in the TLD-resistant evolved populations, or *vice versa*). This cross validation across experimental approaches is important and we hope it convinces the reader that genes with biological significance are being identified here. We thus identified several genes whose products belong to the two previously identified TLD pathways (DNA replication/repair and ROS). Most of the genes here have not been previously identified as modulators of TLD—even the ones whose products fall into pathways previously known to be involved. The identification of over a dozen novel genes involved in the previously unknown contributing pathway of cytoplasmic pH regulation (shown in green purple and pink in Fig 2H&I and S5 Fig) on this list should not be overlooked.

d. Were transposon insertions at the ends of genes considered in the analysis? It is customary to disregard the first and last 5% or so of a gene, because transposon insertions in these locations often don’t inactivate the gene.

We appreciate this suggestion and re-performed the gene-level counts using the truncation suggested as a sanity check. We found that all of the Spearman correlations between the new and old gene-level counts are greater than 0.99. Thus, any effects of masking those regions on our analysis are trivial, and we retain the original analysis (noting that any choice one makes on exactly how much of a gene to mask for this purpose is somewhat arbitrary).

e. Why are ileY and trpL the strongest significant positive and negative significant hits? If these small genes are spurious hits (and it certainly seems that way), the authors should re-evaluate how they do their Tn-seq analysis so that these don't get marked as significant.

We certainly agree with the reviewer that a careful statistical treatment is essential for the interpretation of the data presented here. We note that standard practice in the Tn-seq field generally involves using simple t-tests on the fitness scores obtained from transposon read counts; our treatment in the original manuscript was already substantially <u>more</u> rigorous than those methods, by using proper discrete statistics for the count data. Nevertheless, in order to provide an additional layer of statistical confidence, in the revised manuscript we have added an additional test for high-confidence hits. As noted in the revised text:

> In order to qualify as a high-confidence hit from the Tn-seq screen, the following criteria must all be met: 1. The gene must have at least two separate transposon insertion locations each of which shows an independent significant change in insertion frequency between the conditions being compared (q<0.1 using the R `prop.test` function), and 2. all significant insertions in that gene must have the same direction of fitness effects. These additional criteria help prevent the possibility of PCR jackpotting or other unusual events with single clonal lineages from altering our overall conclusions. Overall, ~60% of genes initially identified in the Tn-seq screen also passed our additional multiple-insertion screening criterion. High confidence hits are noted as such in the tables below and in Supplementary Table 1.

We note that of the key genes identified and discussed in the manuscript, 16/21 of the genes in Table 1 and 18/20 of the genes in Table 2 pass this additional stringent criterion. (The reviewer may also be interested to know that *ileY* and *trpL* do not pass the new criteria). This analysis largely supports our original conclusions, while adding an additional layer of confidence in the key hits that we have identified and built our interpretation on. At the same time, the new tests must necessarily reduce our statistical power; for example, one of the tested genes that was flagged as significant by our standard criterion but not by the more stringent criterion now introduced, *tusC*, did in fact have a substantial fitness effect in follow-up experiments (Panel F of S1 Fig). Particularly keeping in mind that the additional test introduced here must necessarily reduce the power of our analysis, we keep the original statistical treatment for the large-scale analysis performed throughout the manuscript, but include the high-confidence calling as an additional layer of information in both the Tables and Supplement.

2. Given the very large number of genes (~400) that the authors identified as having "significant" phenotypes in their Tn-seq screen, a rigorous statistical treatment is needed to support the inferences drawn by the authors. This is a major issue with the manuscript as written! A few examples below:

a. "As expected, disruptions in genes whose inactivation have previously been shown to sensitize cells to TLD, such as uvrD <21-23>, have significant negative survival scores" Do genes that have been

previously implicated as important for surviving TLD have overall negative scores?

Table 1 shows the 21 significant genes in the previously known pathways of DNA replication/ repair and respiration with their survival scores from our screen. Ten have not been previously implicated in TLD to our knowledge: *cho, cydX, dam, hspQ, ogt, priC, radA, recN, ubiEH*. Of the 3 remaining genes whose inactivation has been previously shown to sensitize cells to TLD, all showed a negative survival score in our screen: *uvrB&D (*-0.88 and -1.1*)*, and *umuC (*-1.34). Note that inactivation of *recJ* was found to alleviate TLD in some studies, but not in others (1-3). Its survival score from our screen was -0.64. We have added this discussion to the manuscript.

b. "…disruptions in genes whose inactivation have previously been shown to enhance survival, such as recO <22, 24, 25>, have significant positive survival scores (Figs 1B-C, S1D Fig)." Again, is this generally true?

Of the 7 remaining genes whose inactivation was previously shown to enhance survival, all had significant positive survival scores in our screen: *cydA&B (*3.76, 3.65)*; ubiG (*3.14)*, recFG (*1.95, 2.85)*, recOR (*1.68, 2.01). We have added this discussion to the manuscript. We also note, as shown in the new panel D in Fig S1, that all of the genes for which prior information in the literature exists regarding TLD survival and for which we observed significant fitness scores, show fitness scores consistent with the previously demonstrated survival effects.

c. "Twenty additional genes with significant fitness effects fall in the previously known pathways of DNA replication and repair, and respiration (Figs 1B-C; Table 1)." Are genes involved in respiration and/or DNA replication and repair significantly enriched in these sets? If one were to pick 400 genes at random some would undoubtedly be involved in respiration and DNA processes.
d. This issue is most pronounced when the authors discuss their most important finding – that genes involved in "the newly identified pathway of pH homeostasis" show TLD phenotypes. There is absolutely no statistical support for this in the manuscript. Where is the data that shows that "genes that produce or import H+ into the cytoplasm, or that lower levels of substrates needed for deacidification systems, enhance survival during thymidine starvation"? Or that "disruptions in genes that consume protons, or produce substrates needed for deacidification systems, exacerbate killing"? Table 2 lists some genes that fall into these categories. But the authors have ~400 genes. There are an unlimited number or "stories" that one could tell by cherry-picking data like this.

We appreciate this suggestion for more robust statistical support of our analysis of the Survival profiling hits. In order to test whether the identified gene categories of interest were enriched in our survival profiling datasets without relying on (potentially spotty) GO term annotations, we defined sets of genes involved in response to acid stress (benzoic acid), DNA damage (nalidixic acid), or oxidative stress (sodium chlorite), using data from the Fitness Browser database (4). We found strong and significant associations of genes that were important in all three categories among those with significant survival scores in our transposon library experiments, with log2 fold enrichments of genes important in the noted stress condition (relative to background) of +1.74 ($p=1.084e-8$; Chi squared test) for acid stress, +0.71 ($p=0.0060$; Chi squared test) for DNA damage stress, and +0.85 ($p=0.0260$; Chi squared test) for oxidative stress. Thus, we observed significant enrichments for functionally relevant genes from all three categories considered here amongst our transposon library hits. We have added these data/analysis to our manuscript.

3. The authors are clearly aware of the literature surrounding TLD in E. coli, and they do an excellent job of summarizing it in the introduction. However, in their analysis they fail to consider previous explanations for their observations. For example, since ROS response and respiration have been shown to play a role in TLD resistance or susceptibility, a novel gene should first be demonstrated not to function by affecting these pathways before a new category of function is proposed. For example, is it possible that ackA plays a role in respiration (or affects its regulation), rather than pH homeostasis?

We appreciate this point. From the 2011 Nichols dataset, a gene deletion in *ackA* makes cells more resistant at pH 4 (fitness Z score of 1.900498) and much more sensitive at pH 10 (-3.283881). Conversely an *ackA* deletion did not make cells more resistant to peroxide (fitness z scores of 0.193895 at .1 peroxide; 0.772461 at .5 peroxide; 0.473169 at 1.0 peroxide; 0.161426 at 1.0 peroxide)

I suggest that the authors revisit and integrate the large body of previous E. coli screens to help contextualize their data. For example, Nichols et al., 2011 (10.1016/j.cell.2010.11.052) performed screens of the E. coli Keio collection under ~300 conditions, including azidothymidine, pH4, and trimethoprim. Interestingly, ackA was the top surviving strain under high azidothymidine.

Thank you for this suggestion. We have now included this in the discussion of our manuscript. See above and below for more use of the Nichols data; we have also made use of more recent Tn-seq experiments to define functionally relevant gene sets, as described above.

4. The authors perform an evolution experiment on two E. coli strains to bolster their findings. I have a two major concerns about this experiment and its interpretation.

First, why were only 2 strains sequenced/discussed in the main text? The authors collected 6 isolated from each of 4 plates in 2 backgrounds. Why not at least sequence one strain from each of the 4 replicates in their experiment? This would give the authors a way to filter random mutations from significant ones and would bolster the credibility of their results.

We did sequence more but due to limited resources and space constraints we chose to focus on the survivors with the greatest effects on survival (and the least growth defects) for this paper – in particular, several of the other evolved lineages showed stronger signs of fitness tradeoffs due to slow growth in non-restrictive conditions.

Second, the authors should be much more clear about what MSD42 is: a MG1655 strain with 699 genes deleted. This hardly justifies the authors should walk back their verbiage on “diverse strains” in the abstract and elsewhere. Out of curiosity, why was this strain chosen instead of something less engineered and more diverse? I personally worry about results from this strain, as many “minimal genome” strains have regulatory loops engineered out of them. Therefore, any results in this strain must be shown to be significant in the wild-type strain as well.

We are now more precise in the MS with the language regarding the genetic backgrounds used. The MDS42 strain was chosen because prophage activation was thought to be a contributing factor in

the killing process (5) and MDS42 has all cryptic prophage deleted (6). Although our starting strain without prophage survives better during the early stages of thymidine starvation (you can see this in our new Fig S7), its degree of death by 24H was very similar to that of the MG1655. This supports observations by previous researchers that substantial death still occurs in the absence of all prophages (2, 5, 7), and provided us with evolutionary data in a genetic background where changes to prophage genes (and silencing) could not possibly play a role.

5. The authors perform RNA-seq on the evolved strains under thymine starvation and then focus on genes with consistent responses – those that are upregulated and the KO is harmful or vice-versa. Is there an enrichment in genes with "consistent" responses vs non-consistent responses? If so, this would support the logic of the authors' strategy. If not, the analysis does not make sense. Also, many previous studies have found little relationship between expression and fitness.

We do see an enrichment in genes that are upregulated in the evolved strains and for which the KO is harmful in all four RNA-seq comparisons:

1. resistant (evolved) starved vs sensitive (parental) starved
2. resistant (evolved) unstarved vs sensitive (parental) unstarved
3. resistant (evolved) starved vs resistant (evolved) unstarved
4. sensitive (parental) starved vs sensitive (parental) unstarved

We now include a figure showing this enrichment in the various comparisons (the new S5 Fig). We do not see the analogous enrichment for genes that are downregulated in the evolved strains and for which the KO is helpful. One explanation is that due to our stringent thresholds for excluding genes with very low read counts, our assay is less sensitive in picking up downregulated genes than upregulated genes; it is also possible that fewer instances of adaptation in our evolution experiments occurred due to reduced expression of harmful genes (i.e. those for which KO is helpful) than did cases of increased expression of helpful genes.

6. The authors' analysis of pHi is intriguing, but I feel like it does not answer the question of whether pHi is causal or not.

a. Do all (or a majority) of genes that confer acid resistance also confer resistance to TLD?

Because there are many genes which contribute to acid resistance, only some of which act directly to raise the cytoplasmic pH and only a smaller subset of which would act quickly enough, under the TLD conditions in our assay, to reduce death, it is not possible to set a strong prediction for how large the overlap should be. We do observe, for example, that 20 out of the 57 genes identified as likely conferring resistance to benzoic acid (in the Fitness Browser dataset described above) are also significant in our fitness screen, representing a significant enrichment over what would be expected by chance.

b. Fig 3 and Fig 4 are extremely difficult to read. Can the authors convert the fluorescence values to pH values? This would both make the figures easy to interpret and would give biologists a sense of how large the reported changes are.

While we appreciate this important issue, getting absolute pH measurements is difficult to establish given time and resource constraints—particularly with the dyes used. Our main goal here was to establish that there are significant relative differences in pH that correlate with survival differences.

c. Since the authors use a fluorescent probe of cytoplasmic pH why is the temporal resolution so bad? The authors should show cytoplasmic pH on a scale of minutes as a function of thymidine starvation. This can be done either using live cell microscopy or flow cytometry. This would relieve a major concern, namely, pH changes occur after >90% killing, as judged by the literature. If the authors kill curves mimic literature findings, pH change cannot be causal

We agree that higher temporal resolution would be very interesting and potentially add but we wanted to make sure we were using the flow cytometry after the lag and once the killing process had begun. In order to determine the time points for these experiments we carried out high temporal resolution kill curves and carefully chose times accordingly. We have now included this information and data in the manuscript. The new S7 Fig shows kill curves for the most sensitive strains before, at, and after the chosen flow cytometry time points. There is around a 1h lag before the cells begin to die. We chose 1.5h, the first time point at which we began to see death, for the first flow cytometry time point. Under our conditions, at the 1.5h thymidine starvation timepoint the *thyA*$^-$ strain in the MDS42 background showed a mean 0.48 $\log_{10}$ reduction, and the *thyA*$^-$ strain in the MG1655 background showed a mean 1.14 $\log_{10}$ reduction. We also chose a time point 1.5h after that to show flow profiles once more killing has taken place and to coordinate with the time point for most of our death assays.

d. An additional strategy that the authors could use to show the importance of acid stress response is to use FACS to sort an isogenic population of thymine starved cells into those with high and low cytoplasmic pH and demonstrate that survival differs between those populations.

We thank the reviewer for this suggestion and have now performed the experiment. In order to test more directly whether the cytoplasmic acidification changes we observe during thymidine starvation cause changes in survival, an isogenic population of *thyA*$^-$ cells was starved of thymidine and stained with both pH sensitive dyes. At 1.5h, which was the earliest time point at which we saw death and significant acidification changes without accompanying significant ROS changes, we sorted cells into the top ~10% and bottom ~10% of the fluorescence distribution, gating stringently so as to only sort cells that are the same size, and then plated them onto high thymidine plates to assess survival differences. Comparing the ratios of survivors in the low to high fluorescent populations, we see that there is significantly increased survival in the low acidification sub-populations compared to analogous sub-populations in the no dye controls (new S10A Fig).

Minor points:

1. L43- "mapped and sequenced at different time points" —as far as I can tell the authors only took one timepoint (3hrs).

We apologize for this confusion. We hope that we are now clearer in the manuscript to be explicit about the time points we took to control for the outgrowths. We mapped and sequenced three time points: one at the start of the selection (0h—without any outgrowth in high thymidine); one at 0h followed by an outgrowth in high thymidine; and one at 3h followed by an outgrowth in high thymidine. As we mentioned above and now are much more diligent to explain in the MS, the outgrowths are necessary

and the 0h timepoints with and without outgrowths allow us to normalize for any outgrowth specific effects that would otherwise bias our survival scores.

2. Fig 1G: L81—were these clean deletions or antibiotic replacements which have an outwardly facing promoter to express downstream gene?

These are clean deletions. Single gene deletions were obtained from the Keio collection (8) and transferred to the *thyA*⁻ strain in the MG1655 background by P1 transduction (9) followed by selection on LB/ kanamycin/thymidine plates. Kanamycin-resistant clones were tested for the clean deletion by PCR, and then cured of the resistance cassette by transformation with the plasmid pcp20 (10) prior to characterization.

3. When discussing the evolution experiments, I think it is important to mention that for the majority of the transfers (43/50) only 2-fold dilutions were performed.

Thank you. We have now added this information.

4. In Fig 3, the "+" on thyA+ is extremely small and difficult to read. Perhaps you could replace thyA+ with WT?

Thank you for this suggestion. We have increased the font of thyA+.

5. Fig 3 Why are the values for the same strains/conditions not consistent across panels? For example, thyA+ is ~4 in panel E and ~0 in panels F, L, and M, despite having the same Y-axis label.

In these figures, all values are offset by the fitted value for the first condition shown, which is thus centered on zero. The critical information is the relative changes in fluorescence between strains. We are now more explicit about this in the figure legend to avoid similar confusion.

Reviewer #3: "Intracellular acidification is a hallmark of thymineless death in E. coli" by A. Ketcham et al. (PLOS Genetics).

In this work the authors investigate the phenomenon of thymineless death (TLD), in which E. coli cells that cannot synthesize the essential compound thymine (thyA cells) are rapidly inactivated (i.e., they die) when asked to grow in a medium lacking either thymine or thymidine. This is in contrast to starvation for most other essential growth components, where the starved cells simply stop growing and enter stasis rather than suddenly dying. This TLD phenomenon has been studied for many decades, and many theories have been proposed to account for the precise mechanism underlying this type of cell death. However, none has appeared to fully account for the TLD. Here, the authors find evidence for yet another mechanistic aspect associated with TLD, namely intracellular acidification, which appears rather early in the death process and may be a major contributing cause to the cell death. The discovery is based on several experimental findings: 1. A saturating search for gene knockouts that either improve or exacerbate TLD reveals several hits of genes controlling cell acidification. 2. The experimental development of TLD-resistant thyA strains shows that these adapted cells have many mutations among which intracellular pH genes are prominent. 3. mRNA expression studies revealing that acidification control genes are specifically affected during TLD. 4. Experimental manipulation of the growth medium that is expected to prevent intracellular acidification (addition of the amino acid arginine) does indeed prevent or diminish TLD. 5. Fluorescent dye indicators are use to directly demonstrate the experimental association of TLD with acidification.

Overall, this is very interesting work. While intracellular acidification is unlikely to be final chapter in understanding TLD, it seems to me that this is an important addition to our current knowledge on the subject. The work seems well performed and interpreted. Publication of this work is recommended.

Here are some issues that the authors need to address:

1. While the Methods section and Figure Legends are chock-full of experimental details (good), the main text suffers as it is in places too succinct and lacks sufficient information for the average reader who may not be fully conversant with all the experimental approaches and lab jargon. The main text should tell a story that can be easily followed by the interested reader, without having to frantically go back and forth between text, Figure Legends, Methods, supplementary Figs and Tables and their associated Legends (as happened to this reviewer, and this is painful). Please improve and clarify your main narrative.

We appreciate this reviewer's thoughtful and helpful appraisal of our work and exposition. We have taken great pains to improve the precision and flow of the main narrative; the changes are too extensive to fully enumerate here, but have mainly involved adding additional contextual information at each point where it would be required.

As one example, let's look at the beginning of the Results section. Here it reads: "A saturated Tn5 transposon insertion library generated in the thyA strain was selected in thymidine-free media and insertions were mapped and sequenced at different time points to look for changes in survival caused by the gene disruptions (Fig. 1A)". This is a solid experimental approach, but the description is not clear. First of all, the authors did not select an insertion library…. The proper description is that they first created an insertion library (see Methods) and subsequently used that library to select mutants affected in TLD. This is a point of confusion, and a clearer description could have saved this reviewer a lot of time. Secondly, when you talk about changes in survival, you need to state (briefly) how you assess survival. Again, unfortunately I had to spend unnecessary time perusing all the details in Methods and Figure Legends to see how you did it. One or two simple sentences in the main text would help the flow. Note that there is no survival curve in the referred to Fig. 1A. I know from previous workers in this field that the survival curves in TLD are somewhat unusual and may contain several informative phases, and the interested reader would like to see what you are talking about.

2. Then the text continues: 'A survival score for each gene was defined as the log2 fold change (LFC) of fragments per million (FPM) at 3 hours (h) versus FPM at 0h". You need to describe this much better. Maybe if you showed some survival curves or reproduced some survival fractions you could guide the reader to how you get to your "survival score". You must make sure that the reader understands this before going on with the rest of the paper. Also, I have no idea what you mean by "fragments per million". What are these fragments? I am sure you are not studying DNA fragmentation, as some have done? The reader cannot go on without being sure what you are talking about. Also, describe what you mean by the positive and negative values for survival scores in the Tables 1 and 2. One could guess, but the authors should be more helpful at this point.

Thank you so much for pointing this out (examples 1 & 2). We have taken pains to clarify and simplify and feel that the exposition is now substantially improved.

3. With regard to the data in Table 3 describing the listing the altered genes in the evolved strains that appear adapted to TLD, there is a discrepancy with the surrounding text. The text describes mutations in oriC (line 100), but I do not see this in the Table. Correct or explain.

We thank the reviewer for pointing out our omission. We have corrected this in Table 3.

4. The description of the genesis of the evolved strains could also use clarification. From the Methods I learn that for each strain (MG1655 and MDS42) four independent lines were started and processed for 50 daily transfer cycles. After 50 transfers the eight cultures were tested for survival, and the best surviving were spread on thymidine-containing medium (line 148). What is meant here? Did you pick the best survivor from MG1655 and the best survivor from MDS42 (i.e., one for each strain)? Please be more clear.

Yes, this is correct. We have added this to the text to be clearer.

Then, you picked six colonies from each best survivor and retested them individually (line 154). From these six, the two top candidates were discussed in the main text (lines 153-155).

The top two candidates are from each background, MG1655 and MDS42. They are not from the same evolved population. We have edited the manuscript to be more precise.

So, to be clear, the two top candidates discussed for each strain were true siblings, i.e., they came from exactly the same evolved culture. This was not clear from the first reading and caused unnecessary confusion. It is important to state this fact separately because it appears that the two top candidates of strain MDS42 shared exactly the same mutations (line 93) except for the atpF gene (line 103). Of course if the two sequenced candidates had been selected from two independent evolved lines this would have been highly unusual.

When we discuss the evolved isolate in the MDS42 background with the atpF mutation, we also discuss one of its siblings that shared all the same mutations except for the *atpF* early stop. These two are siblings from the same evolved population. We now include additional text in the manuscript to clarify the relationship between these two isolates, and their status at *atpF*.

5. The first listed mutation in Table 3 (atpF gene) is listed as Q85*. Does this * refer to some footnote that I did not see? Is this the one with the stop codon? If so, what is the other one? (As an aside, it is interesting that this evolved culture contained two different atpF mutants, ie, the culture was heterogeneous, despite the fact that one survives significantly better than the other, see Fig. 2D).

Yes, the asterisk indicates the stop codon. The sibling has WT *atpF*. We have modified the text to be more clear. We apologize for any confusion this may have caused, and have clarified the point in the revised text

6. I would like to see some more description and explanation of the interesting Figs. 2F and 2G. Can I assume that the 45-degree line represents all the transcripts that do not change in the given comparison?

Yes, this is correct.

Does that also mean that the colored genes represented outside the 45° lines are the only ones that fall outside of this line, or do you list only those genes that you are interested in? (I hope not).

Again, the reviewer is correct. There are other genes that show interesting responses that we do not highlight in 2F&G. Here, as in the rest of this work, we chose to focus on genes in the previously implicated Replication/Repair and ROS pathways (please note that while many of these genes are in previously implicated categories—they themselves have not been previously implicated in TLD), and in the novel category of intracellular pH homeostasis. Table S5 gives a more comprehensive listing of the RNA transcript levels (in Transcripts Per Million) for the parental and evolved strains.

But provide a better narrative for this.

We are now more precise in both the figure legend and the results section.

7. Likewise, Figs. 2H and 2I are interesting but could also use some better explanation. See point 1 above regarding the survival score and FPM, but we also need a better description of the RNA part: LFC of RNA expression (TPM) (transcripts per million?).. A lot of jargon, please explain.

Yes, our transcript counts from our transcriptional profiling experiment were normalized to transcripts per million (TPMs) in order to allow for more direct comparison with the survival profiling data. This was mentioned in the methods but we have also added it to the main text for improved clarity. In order to clarify here and tone down on the bulky jargon, the first time we mention survival scores in this section we remind the reader what they are (LFC of fragments per million). The rest of the time we simply say "survival scores". We hope this helps.

8. Starting on line 173, the authors start using the word "consistent". This is somewhat confusing. I most people's mind this word would mean "reproducible". However, I now realize that the authors mean to say that the effects of genetic modulation and transcriptional responses move in the same expected direction, i.e., consistent with expectation. I suggest other wording, like "corresponding" or perhaps define 'consistent' as 'consistent with expectation' the first few times it is used.

Thank you for this suggestion. We have exchanged "consistent effects" with "concordant effects" which we hope is more precise and will avoid confusion with reproducibility.

1. Fonville NC, Bates D, Hastings PJ, Hanawalt PC, Rosenberg SM. Role of RecA and the SOS response in thymineless death in Escherichia coli. PLoS Genet. 2010;6(3):e1000865.
2. Kuong KJ, Kuzminov A. Stalled replication fork repair and misrepair during thymineless death in Escherichia coli. Genes Cells. 2010;15(6):619-34.
3. Nakayama K, Shiota S, Nakayama H. Thymineless death in Escherichia coli mutants deficient in the RecF recombination pathway. Can J Microbiol. 1988;34(7):905-7.
4. Wetmore KM, Price MN, Waters RJ, Lamson JS, He J, Hoover CA, et al. Rapid quantification of mutant fitness in diverse bacteria by sequencing randomly bar-coded transposons. mBio. 2015;6(3):e00306-15.

5. Khodursky A, Guzman EC, Hanawalt PC. Thymineless Death Lives On: New Insights into a Classic Phenomenon. Annu Rev Microbiol. 2015;69:247-63.
6. Posfai G, Plunkett G, 3rd, Feher T, Frisch D, Keil GM, Umenhoffer K, et al. Emergent properties of reduced-genome Escherichia coli. Science. 2006;312(5776):1044-6.
7. Guarino E, Salguero I, Jimenez-Sanchez A, Guzman EC. Double-strand break generation under deoxyribonucleotide starvation in Escherichia coli. J Bacteriol. 2007;189(15):5782-6.
8. Baba T, Ara T, Hasegawa M, Takai Y, Okumura Y, Baba M, et al. Construction of Escherichia coli K-12 in-frame, single-gene knockout mutants: the Keio collection. Mol Syst Biol. 2006;2:2006 0008.
9. Miller JH. Experiments in molecular genetics. Cold Spring Harbor, N.Y.: Cold Spring Harbor Laboratory; 1972. xvi, 466 p. p.
10. Cherepanov PP, Wackernagel W. Gene disruption in Escherichia coli: TcR and KmR cassettes with the option of Flp-catalyzed excision of the antibiotic-resistance determinant. Gene. 1995;158(1):9-14.
